# A Smartphone App Designed to Empower Patients to Contribute Toward Safer Surgical Care: Community-Based Evaluation Using a Participatory Approach

**DOI:** 10.2196/12859

**Published:** 2020-01-20

**Authors:** Stephanie Russ, Zahira Latif, Ahmarah Leah Hazell, Helen Ogunmuyiwa, Josephine Tapper, Sylvia Wachuku-King, Nick Sevdalis, Josephine Ocloo

**Affiliations:** 1 King's College London London United Kingdom

**Keywords:** patient safety, surgery, smartphone, mobile phone, patient empowerment

## Abstract

**Background:**

MySurgery is a smartphone app designed to increase patient and carer involvement in behaviors that contribute toward safety in surgical care.

**Objective:**

This study presents a pilot evaluation of MySurgery in which we evaluated surgical patients’ perceptions of the app in terms of its content, usability, and potential impacts on communication and safety.

**Methods:**

A participatory action research (PAR) approach was used to formulate a research steering group consisting of 5 public representatives and 4 researchers with equal decision-making input. Surgical patients were recruited from the community using multiple approaches, including Web based (eg, social media, recruitment websites, and charitable or voluntary organizations) and face to face (via community centers). Participants referred to MySurgery before, during, and after their surgery and provided feedback via an embedded questionnaire and using reflective notes.

**Results:**

A diverse mix of 42 patients took part with good representation from 2 “seldom heard” groups: those with a disability and those from a black, Asian, or minority ethnic group. Most were very supportive of MySurgery, particularly those with previous experience of surgery and those who felt comfortable to be involved in conversations and decisions around their care. The app showed particular potential to empower patients to become involved in their care conversations and safety-related behaviors. Perceptions did not differ according to age, ethnicity, or length of hospital stay. Suggestions for improving the app included how to make it more accessible to certain groups, for example, those with a disability.

**Conclusions:**

MySurgery is a novel technology-driven approach for empowering patients to play a role in improving surgical safety that seems feasible for use within the United Kingdom’s National Health Service. Adopting a PAR approach and the use of a diversity strategy considerably enhanced the research process in terms of gaining diverse participant recruitment and patient and public involvement. Further testing with stakeholder groups will follow.

## Introduction

### Background

Optimizing patient safety remains a key priority for health care systems across the world, including the United Kingdom’s National Health Service (NHS). When looking at the frequency of patient safety incidents, surgical care settings typically emerge as the most *risky*, with higher rates of adverse events than other hospital departments or specialties [1-3]. This is likely attributable to the complexity of surgical environments and the risk profile of surgical patients, but it may also reflect higher incident reporting rates by surgical teams.

Numerous tools have been introduced to surgical settings to increase reliability and improve safety, ranging from safety checklists to electronic devices for counting swabs [4,5]. Although these clinical and team-based interventions are critical, the call to deliver more patient-focused interventions in health care is equally important. This is set in the context of an ongoing movement across the NHS and internationally, toward greater patient and public involvement (PPI) and empowerment, working on the concept that, where appropriate, patients should be encouraged to take an active role in the management of their own health and facilitated in participating more meaningfully in their care [6-8]. This focus on patient involvement extends into the world of health care research and evaluation, with patients and the public increasingly being included in all phases of research, from conception, through design and data collection, to dissemination [9]. However, the evidence suggests that PPI efforts, particularly in the world of patient safety, have tended to be atheoretical, exclusive, and tokenistic in nature, with few addressing issues of equality and diversity in their involvement strategies [10-12].

In parallel to the patient empowerment movement, the *industry* of health care is being called to take on another challenge—to better embrace the potential of digital technology for transforming care. In 2016, the Nuffield Trust released a report highlighting the possibilities offered by digital technology and how best to grasp them, putting health care at least a decade behind other industries in terms of incorporation and use of information technology [13]. Smartphones, with their ever-increasing accessibility, have emerged as a key device for communicating knowledge at scale and for improving patient empowerment [14]. Around 85% of the UK population own or have access to a smartphone, including 71% of 55 to 75-year olds, and between 45,000 and more than 300,000 medical health apps are available to download (depending on the platform used) [15-17]. In the context of surgical safety, given their accessibility, smartphone apps could constitute an effective means of mobilizing knowledge to patients about risk and empowering them to play a role in optimizing the safety of their care.

In 2017, the NHS released an online *App Library* with the aim of providing “trusted digital tools to patients and the public to manage and improve their health.” To be listed on the library, an app must meet the NHS quality standards for clinical effectiveness, safety, usability, and accessibility and have evidence to support its use. Therefore, it should be tested with the relevant stakeholder groups [18]. There are currently 76 apps available in the library, falling into the following categories: first aid; living with cancer; mental health and well-being; pre- and postnatal care; welfare and lifestyle; advice, management, and support for long-term conditions; and prescription and appointment management. Although all the apps aim to empower and educate patients in some manner, none of them have a specific focus on involving patients in the effort to improve patient safety. Outside of the NHS, there are apps available that have a greater focus on patient safety. The majority are designed for clinicians or hospital management, for example, digital patient safety manuals, or patient safety solutions for hospitals (eg, the *Patient Safety Solutions* app). Others, designed for patients, have a broad focus on patient education and empowerment (eg, eg, providing information about procedures, conditions, complications, and processes of care; keeping track of upcoming appointments; and informing patients about how to interact and ask questions; eg, the *Empowered Patient*, *Manage my Surgery*, and *Patient Aider* apps) and include some safety-relevant information as part of this. With their broader scope, these apps do not focus specifically on the key evidence-based safety risks present in any particular specialty or pathway.

**Figure 1:**
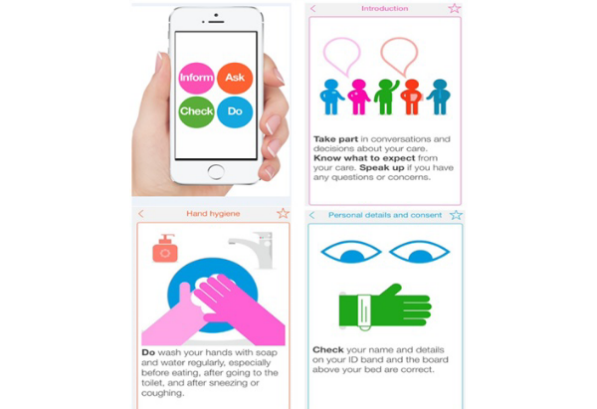
Screenshots from the MySurgery app.

MySurgery is a smartphone app that aims to empower patients to help optimize the safety of their care when having a surgical procedure. The app was developed for the context of the United Kingdom’s NHS and was created by a multidisciplinary team of clinicians, patient safety experts, and patient/public representatives. It has been available for free download on the Apple App Store since 2015 (located under apps for iPhones)—to date, it has been downloaded by more than 6000 people. MySurgery mobilizes evidence around safety in surgery into a format that is easily digestible by patients and their carers. It is an animated app combining simple, jargon-free information and is structured around 10 specific areas of risk to safety: preparing for surgery, personal details and consent, hand hygiene, deep vein thrombosis, falls, pressure ulcers, medications, wound care, nutrition, and going home. For each area of risk, MySurgery provides practical step-by-step advice on the actions that patients and their carers can take—including warning signs to look out for, information to provide, and questions to ask. The app also includes a short introduction (to inform users of its objectives and how it was developed), a *Top 10 Things to Remember* tab (highlighting 10 key behaviors that patients should always aim to engage in), and a link to a survey for evaluating the app (Figure 1) [19]. Until now, formal evaluation of the MySurgery app has not been possible because of funding cessation. However, new funding has recently been secured from the National Institute for Health Research (NIHR), United Kingdom, to evaluate the app over a 3-year program of work. On completion of this work, MySurgery would offer a novel contribution to the NHS App Library and an approach to improving patient safety that is both patient focused and embraces the call for better digital technology solutions to care problems.

### Objectives

This study represents the first phase of this program of work, in which we present results from some pilot testing of MySurgery with surgical patients. To further strengthen the study design, the research was also conducted in partnership with a study being carried out by JO who was funded by the Health Foundation and aimed to understand how best to incorporate diverse PPI into the testing of patient safety interventions. The research objectives are 2-fold:

1. To assess the views relating to MySurgery with a cohort of diverse surgical patients recruited from the community and to understand perceptions of the app, perceived impacts on care and safety, and areas for improvement.
2. To describe and evaluate the approach and impact of incorporating diverse PPI into the project design, planning, and delivery.

## Methods

### Research Approach

The research team was formulated using a participatory action research (PAR) approach, which is appropriate when seeking to solve problems and effect improvement [20,21]. PAR is particularly relevant to the development of PPI as it is a methodology that seeks to empower its research *subjects* and to create more equal partnerships in the research process [22,23]. We used PAR to enable collaborative working within the project by creating a *research steering group*. This consisted of 5 public representatives (AL, HO, JOT, JT, and SW), alongside 4 researchers (SR, ZL, NS, and JO). The public representatives were recruited following attendance at a focus group on the design of the app. They all had some experience of surgery and were selected to represent a diverse range of backgrounds (1 male/4 females; 2 white British/3 black, Asian, or minority ethnic [BAME] group British; 1 with a disability/4 with no disability; and 2 aged <55 years/3 aged >55 years). Steering group members attended face-to-face meetings at 3 key time points across the project and maintained regular communication via email and telephone. In line with PAR, all steering group members were treated as coresearchers and were involved equally in decision making around the design and planning of the project (including development of research tools and recruitment strategy), analysis of the findings (extraction of themes from qualitative data), and write up and review of this manuscript (4 of the 5 public representatives are involved as authors; 1 public representative chose to opt out of authorship but is acknowledged). Owing to time limitations in gaining Disclosure and Barring Service approval, we were not able to involve the public members in collecting the data.

### Study Design

To evaluate MySurgery, the steering group agreed upon a prospective mixed methods design. Both quantitative (survey) and qualitative (reflective notes) methods were used to evaluate patient perspectives of the app with a sample of individuals undergoing surgery recruited from community networks across England. The study ran as a pilot to test the feasibility of the procedure and research tools before future evaluation of MySurgery with a larger cohort of patients. Ethical approval was granted by the King’s College London Research Ethics Review Board (Reference LRS-17/18-5697).

### Participants

Individuals meeting the following inclusion criteria were eligible to participate:

1. Those awaiting surgery between May and June 2018. (Surgery refers to any hospital-based surgical procedure involving an incision, including cesarean sections and tooth extractions, emergency and elective procedures, and day surgery as well as surgery requiring a hospital stay).
2. Those older than 18 years.
3. Those with a good understanding of written English.
4. Those with the capacity to provide informed consent.
5. Those with access to an Apple iPhone or iPad.

Given the narrow time frame and inclusion criteria, we recruited participants using an opportunistic approach via a range of channels, including social media (Facebook and Twitter); bimonthly *calls for research participants* generated by King’s College London; the *Call for Participants* open Web-based platform ; specialist and nonspecialist local community centers; community-based networks of the steering group; and other community voluntary and charitable organizations, including disability groups. We also formulated a structured diversity approach as this objective was central to the study (Textbox 1).

**Participant recruitment: diversity approach.**

Steps taken to achieve a diverse sample:

- By striving for diversity, we would be able to analyze the relationship between participant characteristics and perceptions of the app in a more comprehensive way.
- We used the Equality Act 2010 to inform our conceptualization of diversity [24]. Although all individuals awaiting surgery were eligible to take part, we targeted (via the research advert—Multimedia Appendix 1) 2 groups with protected characteristics who are frequently underrepresented in this type of research—those with a disability and those from a black, Asian, or minority ethnic (BAME) group [12,25]. We hoped that approximately one-third of our participants would have 1 of these characteristics.
- We also aimed to capture diversity in procedure type (all types of hospital-based surgery were included) and geographical location (individuals having surgery at any National Health Service Trust in England were invited to participate).
- A multimethod approach was used to recruit diverse population groups. This included placing our study advert in patient charity newsletters, patient-centered Facebook groups, and specialist and nonspecialist community centers working with local communities across England and with disability groups.
- We monitored diversity of our sample using an Equal Opportunities Monitoring form and via the study questionnaire (see the Outcome Measures section).
- The research team was made up of 2 researchers (JO and ZL), who were culturally competent in developing practices and appropriate, relevant, and sensitive strategies in working with individuals from different cultures and backgrounds.
- We targeted community centers located in areas with a high and diverse BAME population. Individuals working at the centers helped us access specific groups and introduced the study to potential participants. These workers have, over time, developed trusting respectful relationships with their service users and are aware of cultural and religious sensitivities.
- Although all BAME participants recruited to the study spoke fluent English, an amount of code switching [26]—alternating between 2 or more languages during conversations—enabled the researcher to build a rapport with individuals from South Asian backgrounds—this encouraged participation and completion.

### Outcome Measures

Research participants completed the following:

- Equal Opportunities Monitoring (EOM) form: a standardized form used to capture demographic information including ethnicity, gender, age, disability, and sexuality.
- MySurgery feedback questionnaire: a bespoke questionnaire designed to capture attitudes toward MySurgery, which was embedded into the app itself. This captured views regarding the usability, content, impact, acceptability, and appropriateness of the app, alongside suggestions for improvement. It also captured information specific to the individual, including the type of surgery, hospital attended, previous surgeries, and 3 items relating to views about being involved in health care decisions and conversations more generally (Multimedia Appendix 2). Attitude-based questions were answered on a 5-point scale ranging from 1 (completely disagree) to 5 (completely agree). Although the questionnaire was intentionally bespoke and specific to MySurgery, items from Weiner’s standardized implementation outcome measures were included to assess acceptability, appropriateness, and feasibility [27] (Multimedia Appendix 2, items 13, 14, 15, and 20).
- Reflective notes regarding MySurgery: open-ended written reflections regarding patients’ experience of using MySurgery and how it impacted their surgical experience using a bespoke template (Multimedia Appendix 3). The template prompted participants to reflect on how MySurgery affected their relationship with clinical staff and their own involvement in their care, how applicable MySurgery was to them, their thoughts around its content and usability, whether it affected their safety, any improvements they felt were needed, and anything else relevant. (Participants were also given the option to provide these reflections over the phone.)

All members of the research steering group completed a written self-reflection tool as part of the research process to enable critical thinking both about the research process and their involvement in it (findings are highlighted in the Results section).

### Procedure

A study advert (Multimedia Appendix 1) outlining the study aims and inclusion and diversity criteria was distributed via email (to the community networks and organizations), face to face (at community centers), and via social media promotion. Eligible individuals expressed their interest to the research team and received a Participant Information Sheet (that detailed the study procedure), a consent form, and an EOM form to complete by email or in the post. On receipt of the completed forms, they were offered a phone call to explain the procedure further and answer questions. Consenting participants were assigned a unique identifier and were asked to download and refer to the MySurgery app before (preferably before their preoperative appointment), during, and up to 2 weeks following their surgery. They were asked to keep their reflective notes throughout the time they were using the app and to complete the embedded questionnaire following their surgery. On completion and return of the questionnaire and reflective notes, they received a £25 shopping voucher. Data collection took place in May and June 2018.

### Data Analysis

Quantitative analyses were completed using Statistics 24 Software (IBM). Participant characteristics and survey data were summarized using descriptive statistics. To examine the relationship between perceptions of MySurgery and participant characteristics, independent-samples *t* tests were computed—the sample was split according to sex (male/female), disability (yes/no), ethnicity (white/BAME), age (either side of median), length of hospital stay (day surgery/1 or more nights), and general perceptions of patient involvement in hospital care for the 3 relevant questionnaire items (agree/other).

Participant’s reflective notes were divided randomly between members of the steering group for inductive thematic analysis. At the final face-to-face steering group meeting, emergent themes were discussed and cross-checked before collectively agreeing on a list of themes that represented the reflections as a whole. Furthermore, 2 of the researchers (SR and JO) analyzed the steering group self-reflections in a similar manner to extract themes regarding perceptions of the research process and involvement strategy. No software was used for the qualitative analyses.

## Results

### Diversity of Sample

A total of 42 participants took part in the study. Their age ranged from 20 to 70 years (mean age=40 years), with a good split between males and females. Participants were treated at 27 different hospitals across England for 25 different kinds of surgical procedures under a total of 11 surgical specialties, with the majority (31/42, 74%) staying in hospital for a night or longer. Just under half of the participants were from a white background—the remaining were from a BAME background. In addition, 41% (17/42) of the sample reported having a disability (Table 1).

**Table 1:** Summary of participant characteristics (N=42).

Characteristics				Value, n (%)
**Sex**				
	Male			17 (41)
	Female			25 (59)
**Age (years)**				
	18-24			6 (14)
	25-34			11 (26)
	35-44			8 (19)
	45-54			8 (19)
	55-64			5 (12)
	≥65			4 (10)
**Ethnicity**				
	Asian/Asian British			14 (23)
	Black/African/Caribbean/black British			6 (14)
	Other ethnic group (Arab)			2 (5)
	**White (British)**			10 (24)
			White other (Eastern European)	10 (24)
**Disability^a^**				
	Yes			17 (41)
	**If yes, type of disability^b^**			
			Visual	8 (47)
			Hearing	4 (24)
			Mobility	3 (18)
			Learning difficulties	2 (12)
			Other	5 (29)
	No			21 (50)
**Type of surgery**				
	General surgery			8 (19)
	Orthopedics			7 (17)
	Obstetric			5 (12)
	Eye surgery			5 (12)
	Gynecological			3 (7)
	Other			14 (33)
**Previous surgical procedures**				
	None			24 (58)
	1-2			10 (24)
	≥3			7 (17)
**Length of stay**				
	Day surgery			11 (26)
	Overnight or longer			31 (74)

^a^Missing n=4.

^b^5 participants reported more than one disability.

### Perceptions of MySurgery: Questionnaire Data

One participant failed to complete the questionnaire. The sample as a whole was positive about MySurgery (Table 2). A large majority agreed that it was acceptable and appealing, it was useful and informative, it was easy to use, it provided new information, it made them better able to become involved in conversations and ask questions about their care, it changed the way they behaved, it should be recommended to patients, it would make surgery more successful, and that they intended to use it in future. Participants were more unsure about the potential impact the app could have on *safety* per se and whether or not it was applicable to all surgical patients. In general, patients felt the app contained the right amount of information, but of those who disagreed with this statement (7/41, 17%), all but 1 felt it contained too little information. A third (14/41, 34%) of the sample encountered technical difficulties when using the app, which were all related to problems downloading it onto an iPad. Over half of the sample (25/41, 60%) said that they would support an option to incorporate audio into the app and availability of an *easy-read* version, and more patients (30/41, 71%) said that they would like to know how to access support in using MySurgery.

**Table 2:** Summary of survey responses for the 18 items relating to perceptions of MySurgery (N=41).

Questionnaire item	Completely disagree, n (%)	Disagree, n (%)	Neither agree nor disagree, n (%)	Agree, n (%)	Completely agree, n (%)
MySurgery meets my approval	0 (0)	4 (10)	9 (21)	24 (57)	4 (10)
MySurgery is appealing to me	0 (0)	3 (7)	8 (19)	26 (62)	4 (10)
MySurgery seems applicable to all surgical patients	0 (0)	13 (31)	18 (43)	9 (21)	1 (2)
I found MySurgery useful and informative	1 (2)	2 (5)	5 (12)	29 (69)	4 (10)
MySurgery provided me with new information	1 (2)	4 (10)	4 (10)	31 (74)	1 (2)
The content of MySurgery is appropriate	1 (2)	3 (7)	10 (24)	24 (57)	3 (7)
I felt the right amount of information was provided	2 (5)	5 (12)	9 (21)	23 (55)	2 (5)
MySurgery was easy to use	1 (2)	3 (7)	6 (14)	29 (69)	2 (5)
I found it difficult to navigate through the information on the MySurgery app	4 (10)	19 (45)	11 (26)	6 (14)	1 (2)
MySurgery made me feel better able to ask questions	0 (0)	4 (10)	5 (12)	31 (74)	1 (2)
MySurgery will help patients to become more involved in conversations around their care	0 (0)	2 (5)	7 (17)	31 (74)	1 (2)
Using MySurgery changed the way I behaved	1 (2)	1 (2)	7 (17)	30 (71)	2 (5)
It is unrealistic to expect patients to use the information provided in the app	2 (5)	9 (21)	22 (52)	7 (17)	1 (2)
MySurgery should be recommended to all patients awaiting surgery by their doctor or nurse	0 (0)	2 (5)	10 (24)	24 (57)	5 (12)
I would recommend MySurgery to other people having surgery	1 (2)	1 (2)	8 (19)	25 (60)	6 (14)
Using MySurgery would make me safer when having an operation	1 (2)	3 (7)	15 (36)	20 (48)	2 (5)
Using MySurgery would make surgery more successful	0 (0)	2 (5)	2 (5)	25 (60)	12 (29)
I intend to use MySurgery for any future surgeries I have	0 (0)	4 (10)	6 (14)	29 (69)	2 (5)

### Relationship With Patient Characteristics

There were no significant differences in perceptions of MySurgery according to sex, participants’ age, ethnicity, or length of stay.

Those with a disability were significantly less likely to agree that MySurgery was easy to use (t_39_=2.22; *P*<.04) and that they would recommend it to others having surgery (t_39_=2.45; *P*=.02).

Those who had experienced at least one previous operation were significantly more positive about MySurgery for 5 of the 18 questionnaire items that related to perceptions of the app than those who had not had a previous operation. They were more likely to approve of it (t_39_=2.27; *P*=.03), to find it appealing (t_39_=2.48; *P*=.02), to feel it contained the right amount of information (t_39_=2.87; *P*<.001), suggest that it should be recommended to all patients (t_39_=2.69; *P*<.001), and suggest that it would make them feel safer (t_39_=2.56; *P*=.01).

Participants’ views about being involved in conversations and decisions around their care in general had the most striking impact on their views of MySurgery. Those who agreed that they were confident to play an active role in conversations around their care (n=15), that they could help to reduce errors by being involved (n=23), and that it is best for patients to be involved in decisions around treatment and safety (n=30) were significantly more positive about the app for a large number of questionnaire items than those who disagreed or neither agreed nor disagreed with these statements (*t*s range: 1.0-4.3; *P*s range <.001-.04).

### Perceptions of MySurgery: Participant Reflective Notes

Overall, 8 themes were extracted from the analysis of participants’ reflections (Table 3).

#### Content/Usability of MySurgery

The app content was deemed to be useful and pitched at the right level. Many commented on how it served as a useful reminder of things they needed to do and helped them to prepare for their surgery. Some picked out particular sections they had found helpful, for example, hand hygiene. However, there were some conflicting comments—for example, although many found it refreshingly simple, others commented that is was too basic and they wanted more information. In terms of usability, comments again were largely positive, for example, easy to use, nice graphics, and easy to navigate. All negative comments in this respect were related to problems with downloading the app.

#### Empowerment and Involvement

This was a significant and positive theme. Most felt that MySurgery had empowered them in some way and had enabled them to become more involved in conversations and decision making relating to their care. Predominantly, this was reflected in feeling that they were able to prepare and ask more relevant questions and that the app promoted self-agency, proactivity, and confidence. Some commented on how it helped them to prepare before consultations when they were less stressed and distracted.

#### Encouraging Self-Care

A significant number of patients commented on how the app had provided them with new information that enabled them to care better for themselves, particularly after surgery, for example, wound care, hygiene, and nutrition. Requests were made for more of this information (eg, in relation to returning to work).

#### Improved Emotional Well-Being

A large majority of patients felt that using MySurgery had helped them to cope emotionally, for example, promoting confidence, providing a sense of security, reducing anxiety and worry, and making them feel less alone.

#### Patient Involvement in Safety

This was an interesting theme given the objective of MySurgery. Almost half (20/41, 48%) of the sample was unsure about the concept of *safety*—assuming safety meant better surgical outcomes rather than reduced risk of error. Others were unsure about how an app could affect safety and stated explicitly that keeping patients safe was the responsibility of the clinical staff alone. These views were not specific to patients with any particular demographic profile, that is, ethnicity/age/disability. The remaining patients were able to demonstrate specifically how the app might enhance safety and provided examples, for example, increasing awareness of risk, of complications to look out for, and of the role patients can play in flagging inconsistencies in care. One patient gave an example of how the app triggered them to identify a drug error.

#### Diversity and Inclusivity

Some of the BAME and Eastern European participants and those with a disability highlighted how MySurgery was not currently ideally suited for certain groups or suggested adaptations that would improve the utility of the app for them. For example, comments were made around the difficulties those with mental health problems or learning difficulties might have in accessing the app or working with the information provided. Others highlighted the need to tailor the app to be more inclusive of different cultures, for example, to refer to different cultural diets or alternative therapies and to make MySurgery available in different languages. Others highlighted a lack of information around vegan and vegetarian diets or information for those with certain allergies. Some highlighted where certain elements of the content were not relevant for particular procedures, for example, dental surgery, eye surgery, or other basic procedures.

#### Improvements

More generally, participants were forthcoming with various suggestions for improving MySurgery. Some related to technological issues with the app, for example, improving the ease of downloading on to iPads and making MySurgery available on Android devices. Others related to improving the content, for example, adding links to more specific procedure-related information or websites, providing more detail in general, and adding audio/videos to make it more interactive.

#### Unintended Consequences

Although this was not a strong theme in the data, it was considered important to be aware of the potential for unintended consequences of MySurgery. A small minority of patients (3/41, 7%) found the app anxiety provoking, in that it made them aware of risks they had not previously considered and increased their level of worry. One other patient commented that staff appeared irritated with the number of questions being asked, raising the question as to whether MySurgery could create tension with staff in certain circumstances.

**Table 3:** Themes extracted from reflective notes with illustrative quotes (N=42).

Theme	Illustrative quotes
Content and usability: comments relating to the content of MySurgery, including its acceptability and appropriateness, and comments relating to ease of use, interface, or technological issues	“The medical language makes no sense. MySurgery app explained medical language and I understood it” “The dos and don’ts list (top 10 things to remember) was incredibly helpful alongside deconstructing myths surrounding surgery in general which I liked” “It reminds you of all the things the doctors tell you but you forget to do” “It was well laid out, nice visually and easy to navigate through the steps” “My wife and I are old. We’ve never used a phone app but used this one because it was easy to use”
Empowerment and involvement: influences on patients’ ability to become involved in their care and their feelings around this	“I was proactive and I think the clinicians thought I was a good patient” “I was able to share information with the midwives who found the app useful too” “It made me feel more positive about asking questions” “I came prepped with questions and was able to get these answered” “I felt empowered by having the information earlier when I was less stressed” “Before my preop appointment I was able to use the app to help me make decisions and consider certain aspects of my surgery and recovery” “I sometimes go into my appointments with a list of question I don’t ask. Doctors think I’m complaining. The app helped with these feelings.”
Encouraging self-care: reflections around how the information within MySurgery influenced the ability to care for one’s self before or after surgery	“After surgery I learnt how to care for the wound” “Gives good information about preparing for surgery and caring after surgery” “It made me less agitated about looking after my surgical wound- in terms of how to take care of it” “I think the app was particularly useful for aftercare of an operation and this could be expanded upon”
Improved emotional well-being: positive reflections about how using MySurgery made patients feel	“It did help with my confidence and general sense of security as I was quite scared to be on my own in hospital” “Living alone and having access to support after surgery were important for me. With the app I did not feel alone” “The app was helpful as it made me feel in control of what was happening” “I had major surgery and this app helped to calm my nerves as I knew what to expect” “I found the information very useful and it made me feel less worried about what was going to happen”
Patient involvement in safety: comments around the impact of MySurgery on surgical safety, whether it was conceptualized to be related to safety and in what way	“Surgeons are involved with safety, not patients” “Doctor and nurse should check everything, that’s not my job” “Safety up to the doctors not patient. Doctors/nurses are paid to make sure the operation is carried out properly” “I did not know about all the risks after surgery – the app helped me to understand these” “Yes it made you aware that your recovery is in your hands as well as the surgeon’s” “It made me think to check everything because hospital very busy” “It made me more cautious towards potential complications that I could keep an eye out for” “Of course it improved safety. Patients can’t expect staff to do everything, the NHS is over-burdened as it is” “When patients are involved in their surgery, they can help to improve safety. Things like preventing infections”
Diversity and inclusivity: reflections around areas or groups to whom MySurgery may be less accessible, acceptable, or relevant	“It is a good app but it needs to be made more user friendly for people with learning difficulties or mental health problems” “Maybe needs to be in different languages” “More dietary information for vegans” “What about people who don’t have access to the internet?” “More detail in the nutrition section for ethnic backgrounds is needed”
Unintended consequences: potential unintended consequences of using MySurgery that may conflict with the objectives of the app	“Staff felt I (was) asking too many questions but I felt happy to ask them after using App” “I normally worry about everything and this app made me worry if my surgery will be successful. I come from another country where we leave everything to the doctors. As patients we do not get involved in our care. I like this” “I did not like it, information made me scared to have operation”
Improvements: suggestions for improvements and additions that could be made to MySurgery, whether in terms of content, design, or usability	“I think it would be really good to eventually have some sections addressing specific types of surgery, and/or resources linked to the hospital/Trust or location were the surgery is taking place” “Maybe it could give information on the most common operations” “Would like to see the ability to add individual bullet points in a page rather to my favourites list as opposed to the whole page” “Signpost to other websites/services for more information” “Maybe if you added videos to make it interactive and more person centred” “There needs to be more detail—it is very basic” “Please make it available for other smartphones”

### Steering Group Self-Reflections

Key themes that emerged from the self-reflections of the steering group were as follows:

- The importance of using a research strategy based on coproduction that allowed public members to feel like equal partners in the research and to gain key research skills and knowledge.
- The fact that having diverse public involvement allowed a much richer contribution of perspectives and ideas to emerge in the research.
- Having strong public involvement meant that there was an important and critical challenge to the formal expertise of the lead researchers, which was invaluable in shaping the design and implementation of the work and cross-checking the validity of the research strategy.
- A reflective tool was useful in enabling people to think more about their own subjectivity either as a researcher, layperson, or current patient or as someone from a particular ethnic or professional background and how this might influence the research process.

## Discussion

### Principal Findings

MySurgery is a novel smartphone app that works on the premise of educating and involving patients and their carers in behaviors that are known to contribute toward surgical safety and to increase resource in the health care system for preventing safety incidents. It has the potential for inclusion in surgical care pathways in the NHS and the NHS App Library should the data be there to support its use and effectiveness.

We gathered views regarding MySurgery from a diverse group of patients undergoing a range of surgical procedures in hospitals across England. Using a survey combined with reflective notes, we were able to conduct a critical analysis of participants’ perceptions of the app that can inform improvements to the app itself and areas to probe in the next phase of evaluation. Overall, the feedback was positive, and there was a strong sense of support for MySurgery. It was reported to act as a useful reminder of steps to take in preparing for surgery, it empowered patients to engage in conversation with clinicians and prompted questions to ask, it promoted confidence in caring for surgical wounds, it reduced anxiety about the surgical process, it was reported to be easy to use, and it would be recommended to others in its current form by 76% (31/41) of participants. This feedback, particularly in relation to promoting greater patient empowerment, shows that the perceived impact of MySurgery was in line with the objectives of the app.

Those who had undergone previous surgery were more positive about MySurgery in a number of respects. Perhaps having more knowledge and experience of the surgical process, and reflecting from previous as well as current experience, allowed participants a greater appreciation of the potential virtues of the app. However, the strongest predictor of perceptions was individuals’ views about whether they personally felt confident to be involved in conversations around their care and whether they thought patients *should* be involved in health care conversations and decisions making. Those who felt uncomfortable to engage in health care conversations and did not feel that they could have an impact on the occurrence of error were less supportive of the app (although not entirely unsupportive). This suggests that MySurgery will likely be more appealing to those who already feel that they are able to play a role in their care or think it is appropriate to do so. It also highlights how some patients are unclear about the role patients *can* play or may need more support becoming involved.

Reflections about the impact of MySurgery on safety were also interesting. Almost half of the patients were unclear what was meant by *safety*, assuming we were referring to procedural success as opposed to reduction in risk of error, and the same proportion felt strongly that safety was a matter for clinicians alone. Many recognized that they had become more involved in safety-relevant behaviors after using MySurgery (eg, checking medication and providing a more thorough history), but they did not describe this in terms of *safety* per se. This seems to reflect both a lack of awareness about safety issues in health care in general and a lack of insight into the role a patient might be able to play, which highlights a challenge for those working in the area of patient empowerment in terms of how best to promote the scope for patient involvement in safety and how to encourage the formation of working partnerships between clinicians and patients. Interestingly, in this sample, we did not find any difference in the willingness to become involved in safety between those from different backgrounds (in terms of ethnicity and disability), which is an important finding to explore further, given how often these groups are excluded from involvement processes in quality and safety.

There was evidence to suggest that MySurgery may be currently less suited to certain patient groups, which was highlighted by the theme of diversity and inclusivity. Those with a disability reported more issues in using the app and were a key group that would be less likely to recommend it to others. The suggested additions of audio, videos, signposting, easy-read versions, and support in using the app may help here. Likewise, although ethnicity was not related to views of the app in general, there were some limitations identified with regard to a requirement for more nutrition-related information for different cultures (as well as those with restricted diets) and availability of the app in different languages. Finally, a few suggested that MySurgery is likely to be less relevant for certain procedures (eg, dental, eye, or minor procedures with fewer inherent risks).

When introducing a new intervention, it is critical to analyze the potential for unintended consequences. Although MySurgery was deemed to have a beneficial emotional impact by most, 3 participants reported that it made them feel *more* anxious about their surgery as they became more aware of potential risks/complications. The content of MySurgery was designed to be intentionally nonanxiety provoking; however, this should be explored in future evaluations. One participant inferred that clinicians might be resistant to patients asking more questions, and it would be interesting to explore this further with clinicians. Both potential issues might also highlight areas that will need to be addressed during implementation.

### Strengths, Limitations, and Next Steps

A key strength of the project was the PAR approach taken in setting up the research steering group. Through a process of self-reflection, we were able to demonstrate a number of beneficial impacts, including a richer contribution of perspectives to project design and data analysis and validation of the approach from a more diverse group of decision makers. A second strength was the diversity achieved in the sample. By implementing a structured diversity approach, we were able to include good representation (17/42, 41% and 22/42, 52% of the sample, respectively) from 2 groups with protected characteristics (disability and BAME background) who are often described as *seldom heard* in health care research. As a result, we have identified steps that can be taken to adapt the app to make it more suitable to these groups.

In terms of limitations, this was a pilot sample totaling 42 participants. Accordingly, although the sample was diverse, the subgroups were relatively small, and formal cross-groups analyses will not be definitive as they lack statistical power. Therefore, any subgroup effects reported in this study (eg, with regard to ethnicity or disability) should be interpreted with caution and will be followed up in the next phase of the study with a larger patient cohort. We did not assess the impact of socioeconomic status or geographic region, which may impact the ability to access the app; this should also be addressed going forward. In addition, we did not assess actual use of MySurgery in real time (eg, via observation) because of the methodological challenges inherent in doing so, and we did not assess clinicians’ views of the app at this stage.

Next steps are to evaluate MySurgery with surgical staff and a larger cohort of patients, using themes identified here to refine the research questions and outcome measures and to test subgroup effects more robustly. Feedback from this pilot study and the next phase of the research will then be used to make improvements to the app. We will also be more formally exploring the design and interface of MySurgery, focusing on user experience, with Mindwave Ventures—UK experts in the design and implementation of digital technology solutions to health care problems. Following improvements to the app, the final objective will be to work with NHS Trusts to explore the best approach to implementing MySurgery into surgical care pathways and to include it on the NHS Apps Library.

### Conclusions

MySurgery is a smartphone app that brings together efforts to empower patients and their carers to become involved in their care and, specifically, to play a role in enhancing surgical safety, with the movement to embrace the potential of digital technology to transform health care. Our findings show that MySurgery has particular potential to empower patients to become involved in health care conversations, shared decision making, and safety-related behaviors. Adopting a PAR approach and the use of a diversity strategy also considerably enhanced the research process in terms of gaining diverse participant recruitment and PPI in the process. Further research is needed to explore why some patients felt less comfortable with their involvement in safety issues and to look more closely at how particular groups of patients, such as those with disabilities, can be empowered through use of the MySurgery app.

## Appendix

Multimedia Appendix 1 Study advert.

Multimedia Appendix 2 MySurgery feedback questionnaire.

Multimedia Appendix 3 Template for reflective notes.

## Abbreviations

BAME: black, Asian, or minority ethnic
EOM: Equal Opportunities Monitoring
CLAHRC: Collaboration for Leadership in Applied Health Research and Care
NHS: National Health Service
NIHR: National Institute for Health Research
PAR: participatory action research
PPI: patient and public involvement

## References

[1] de Vries EN, Ramrattan MA, Smorenburg SM, Gouma DJ, Boermeester MA (2008). The incidence and nature of in-hospital adverse events: a systematic review. Qual Saf Health Care.

[2] Baines RJ, Langelaan M, de Bruijne MC, Wagner C (2015). Is researching adverse events in hospital deaths a good way to describe patient safety in hospitals: a retrospective patient record review study. BMJ Open.

[3] Rafter N, Hickey A, Conroy RM, Condell S, O'Connor P, Vaughan D, Walsh G, Williams DJ (2017). The Irish National Adverse Events Study (INAES): the frequency and nature of adverse events in Irish hospitals-a retrospective record review study. BMJ Qual Saf.

[4] Ramsay G, Haynes AB, Lipsitz SR, Solsky I, Leitch J, Gawande AA, Kumar M (2019). Reducing surgical mortality in Scotland by use of the WHO Surgical Safety Checklist. Br J Surg.

[5] Wakeman D, Langham MR (2018). Creating a safer operating room: groups, team dynamics and crew resource management principles. Semin Pediatr Surg.

[6] Jenerette CM, Mayer DK (2016). Patient-provider communication: the rise of patient engagement. Semin Oncol Nurs.

[7] Francis R (2013). Report of the Mid Staffordshire NHS Foundation Trust Public Inquiry: executive summary.

[8] (2014). International Alliance of Patients' Organizations.

[9] (2018). Health Research Authority.

[10] Ocloo J, Garfield S, Dawson S, Dean Franklin B (2017). Exploring the theory, barriers and enablers for patient and public involvement across health, social care and patient safety: a protocol for a systematic review of reviews. BMJ Open.

[11] Ocloo J, Matthews R (2016). From tokenism to empowerment: progressing patient and public involvement in healthcare improvement. BMJ Qual Saf.

[12] Dawson S, Campbell SM, Giles SJ, Morris RL, Cheraghi-Sohi S (2018). Black and minority ethnic group involvement in health and social care research: a systematic review. Health Expect.

[13] Imison C, Castle-Clarke S, Watson R, Edwards N (2016). The Nuffield Trust.

[14] Calvillo J, Román I, Roa LM (2015). How technology is empowering patients? A literature review. Health Expect.

[15] (2017). Deloitte.

[16] Deloitte.

[17] (2017). research2guidance.

[18] National Health Services.

[19] Davenport F (2014). Imperial College London.

[20] Reason P, Bradbury H (2008). The SAGE Handbook of Action Research Second Edition.

[21] Meyer J (2000). Qualitative research in health care. Using qualitative methods in health related action research. Br Med J.

[22] Bradbury H, Lifvergren S (2016). Action research healthcare: focus on patients, improve quality, drive down costs. Healthc Manage Forum.

[23] Tossavainen PJ (2017). Co-create with stakeholders: action research approach in service development. Action Res.

[24] (2010). UK Legislation.

[25] Beresford P (2013). Beyond the Usual Suspects: Towards Inclusive User Involvement.

[26] Ballard R, Ballard R, Banks M (1994). Introduction: the emergence of Desh Pardesh. Desh Pardesh: The South Asian Presence in Britain.

[27] Weiner BJ, Lewis CC, Stanick C, Powell BJ, Dorsey CN, Clary AS, Boynton MH, Halko H (2017). Psychometric assessment of three newly developed implementation outcome measures. Implement Sci.

