# Supplementary material for: A Smartphone App Designed to Empower Patients to Contribute Toward Safer Surgical Care: Community-Based Evaluation Using a Participatory Approach
Source: JMIR Mhealth Uhealth. 2020 Jan 20;8(1):e12859. doi: 10.2196/12859 (PMC6997920; doi:10.2196/12859)
Supplement: Multimedia Appendix 1 [file mhealth_v8i1e12859_app1.pdf]

Version 3: 23.03.18

ARE YOU DUE TO HAVE SURGERY BEFORE MID-JUNE 2018?

Help improve surgical care in the NHS

This study is sponsored by King's College London and has received full ethical approval from the King's College London Research Ethics Committee

Participants will receive a £25 voucher as a gesture of thanks for their time

Background

Ensuring patients get the best quality and safest possible care is a major priority for NHS England. Being at the centre of their care, patients having surgery and their families/carers can help NHS staff to deliver safer care and to ensure the best possible surgical experience, if they are appropriately informed.

Project aims

The aim of this research project is to explore the use of a smartphone app, called MySurgery (free to download on the App Store), which informs surgical patients and their families/carers of the actions they can take to help improve experience, safety and outcomes when having a surgical procedure.

How can you help?

- **Current opportunity:** We are inviting 50 individuals who are due to have surgery between May and mid-June 2018 to participate in this research. By surgery, we mean any hospital-based surgical procedure that involves an incision (including caesarean sections and tooth-extractions, emergency and elective procedures, and day-surgery as well as surgery requiring a hospital stay). We are asking individuals who fit this criteria to download the MySurgery smartphone app prior to their pre-operative appointment, to refer to it across their surgery, and then to provide us with feedback on their use of the app via a questionnaire within the app and by completing a reflective diary or interview. The app is purely informative and does not affect the process of care in any way. Views about MySurgery will help to inform improvements to its content, design and delivery, such that it can be built into care pathways to benefit surgical patients across the NHS.
- **Essential Criteria**: In addition to the above criteria, in order to participate in this research you must be over 18 years of age and be able to understand written English (as MySurgery is currently only available in English). You must also have access to an iPhone or iPad so that you can download the app (unfortunately it is currently unavailable on Android devices).
- **Desired criteria**: We are particularly interested in including individuals from a Black, Asian and Minority Ethnic group background and those with a disability. However, everyone is encouraged to respond to this invitation regardless of their background.

Contact Information

If you are interested in becoming involved in this research, please contact the research team using the email address below. You will be provided with further details at this stage.

Email: zahira.latif@kcl.ac.uk
