# Supplementary material for: A Smartphone App Designed to Empower Patients to Contribute Toward Safer Surgical Care: Community-Based Evaluation Using a Participatory Approach
Source: JMIR Mhealth Uhealth. 2020 Jan 20;8(1):e12859. doi: 10.2196/12859 (PMC6997920; doi:10.2196/12859)
Supplement: Multimedia Appendix 2 [file mhealth_v8i1e12859_app2.pdf]

NHS
National Institute for Health Research

King's College London

MySurgery: Patient Feedback Questionnaire

The primary aim of this questionnaire is to collect feedback on your views of the MySurgery app so that we can understand what people think about it and how it might be improved. We will also be asking some general questions about yourself (which are optional) and your views about receiving treatment in hospital settings. We anticipate it will take no more than 15 minutes to complete the questions.

Please answer the following questions which are designed to collect some more information about you:

1. What type of operation did you have? ____________
2. Where did the surgery take place? i.e. at which hospital? ________________________
3. Which of the following applies to you?
   a. I had day-surgery
   b. I stayed in hospital over-night or longer
4. Did you inform your clinical team that you were using the MySurgery app? Yes/No
5. If yes, was this received positively by your clinical team? Yes/No

   Please use this space to elaborate on your answer

   ____

   ____

6. Please select your sex: Male/ Female
7. How old are you? _______
8. How many previous surgical procedures have you had? (this includes tooth extractions in a hospital setting and caesarean sections)
   a. None
   b. 1-2
   c. 3 or more
9. Are you confident in using smartphone apps (please select): Yes / No

Please read each of the following statements carefully which relate to your views about attending hospital for treatment and about being involved in your care. Please select the answer for each statement that best reflects your own views – there are no right or wrong answers

To what extent do you agree or disagree that…?
Please tick one box only for each statement

1 -----------2-- ------- 3 --------- 4 ---------- 5

		Completely disagree	Dis-agree	Neither agree or disagree	Agree	Completely agree
10..	I feel confident to play an active role in conversations in hospital	1	2	3	4	5
11.	I think that I could help to reduce errors in my care by being more involved	1	2	3	4	5
12.	It is best for patients to be involved with health professionals in decisions about treatment and safety"	1	2	3	4	5

Please read each of the following statements which relate to your attitudes towards the MySurgery app. Please select the answer for each statement that best reflects your own views – there are no right or wrong answers

To what extent do you agree or disagree that…?
Please tick one box only for each statement

		1 Completely disagree	2 Dis-agree	3 Neither agree or disagree	4 Agree	5 Completely agree
13.	MySurgery meets my approval	1	2	3	4	5
14.	MySurgery is appealing to me	1	2	3	4	5
15.	MySurgery seems applicable to all surgical patients	1	2	3	4	5
16.	I found MySurgery useful and informative	1	2	3	4	5
17.	MySurgery provided me with *new* information	1	2	3	4	5
18.	The content of MySurgery is appropriate	1	2	3	4	5
19.	I felt the right amount of information was provided in the app*	1	2	3	4	5
20.	MySurgery seems easy to use	1	2	3	4	5
21.	I found it difficult to navigate through the information on the MySurgery app					
22.	MySugery made me feel better able to ask questions	1	2	3	4	5
23.	MySurgery will help patients to become more involved in conversations around their care	1	2	3	4	5
24	Using MySurgery changed the way I behaved	1	2	3	4	5
25	It is unrealistic to expect patients to use the information provided in the app	1	2	3	4	5
26	MySurgery should be recommended to all patients awaiting surgery by their doctor or nurse	1	2	3	4	5
27.	I would recommend MySurgery to other people having surgery	1	2	3	4	5
28.	Using MySurgery would make me feel safer when having an operation	1	2	3	4	5
29.	Using MySurgery would make surgery more successful	1	2	3	4	5
30.	I intend to use MySurgery for any future surgery I have	1	2	3	4	5

*(If disagree or completely disagree): In my opinion the app contained:

a. Too much information
b. Too little information
c. Other____

31. I encountered technical difficulties when using MySurgery (please select): Yes / No

If yes, please provide details of the difficulties experienced

____

____

39. Would you find any of the following adaptations to MySurgery useful? (please select all that you would find helpful)

a. An option to include audio on the app
b. An Easy read version of the app
c. Information for where to find support in using the app
d. Other_______________________________________

Please use this space to expand upon any of your answers or to provide further comments about the MySurgery app, including any information you think needs to be added/removed, or any improvements that could be made.

THANK YOU VERY MUCH FOR YOUR HELP!
