# Supplementary material for: A Smartphone App Designed to Empower Patients to Contribute Toward Safer Surgical Care: Community-Based Evaluation Using a Participatory Approach
Source: JMIR Mhealth Uhealth. 2020 Jan 20;8(1):e12859. doi: 10.2196/12859 (PMC6997920; doi:10.2196/12859)
Supplement: Multimedia Appendix 3 [file mhealth_v8i1e12859_app3.pdf]

Participant ID No:

Reflective Notes

Please feel free to write as much you like and use extra sheets if you need them.

How do you feel the MySurgery app impacted on your experience of having surgery?

Tip: Please reflect on your complete surgical journey (before, during and after surgery) and think about how the app influenced you (if at all). For example, as part of this, please think about how the app influenced your relationship with the clinical staff, whether it prompted you to ask any questions or provide information, and how applicable you felt the content of the app was to you. In your reflections, please think about any aspects of your background (e.g. race/ethnicity, gender, disability age or anything else you wish to highlight).

Participant ID No:

What are your thoughts about the content and usability of the app?
Tip: Did the content make sense? Was it helpful or unhelpful? Was there anything missing? Was it easy to use? Which aspects did and didn’t work? Would you be confident to use the app?

Did the app enable you to be better involved in your surgical care? If so, how? If not, why?

Participant ID No:

Did the app improve the safety of the surgical procedure you undertook? If so, how? If not, why?

Was there anything about the app that empowered or disempowered you in your care?

Is there anything you would improve about the app and what would this be?
